# Supplementary material for: A model for background selection in non-equilibrium populations
Source: bioRxiv. 2025 Sep 16:2025.02.19.639084. Originally published 2025 Feb 20. Preprint. [Version 5] doi: 10.1101/2025.02.19.639084 (PMC11870586; doi:10.1101/2025.02.19.639084)
Supplement: 1 [file NIHPP2025.02.19.639084V5-supplement-1.pdf]

bioRxiv preprint doi: https://doi.org/10.1101/2025.02.19.639084; this version posted September 16, 2025. The copyright holder for this preprint (which was not certified by peer review) is the author/funder, who has granted bioRxiv a license to display the preprint in perpetuity. It is made available under aCC-BY 4.0 International license.

Supplemental Figures

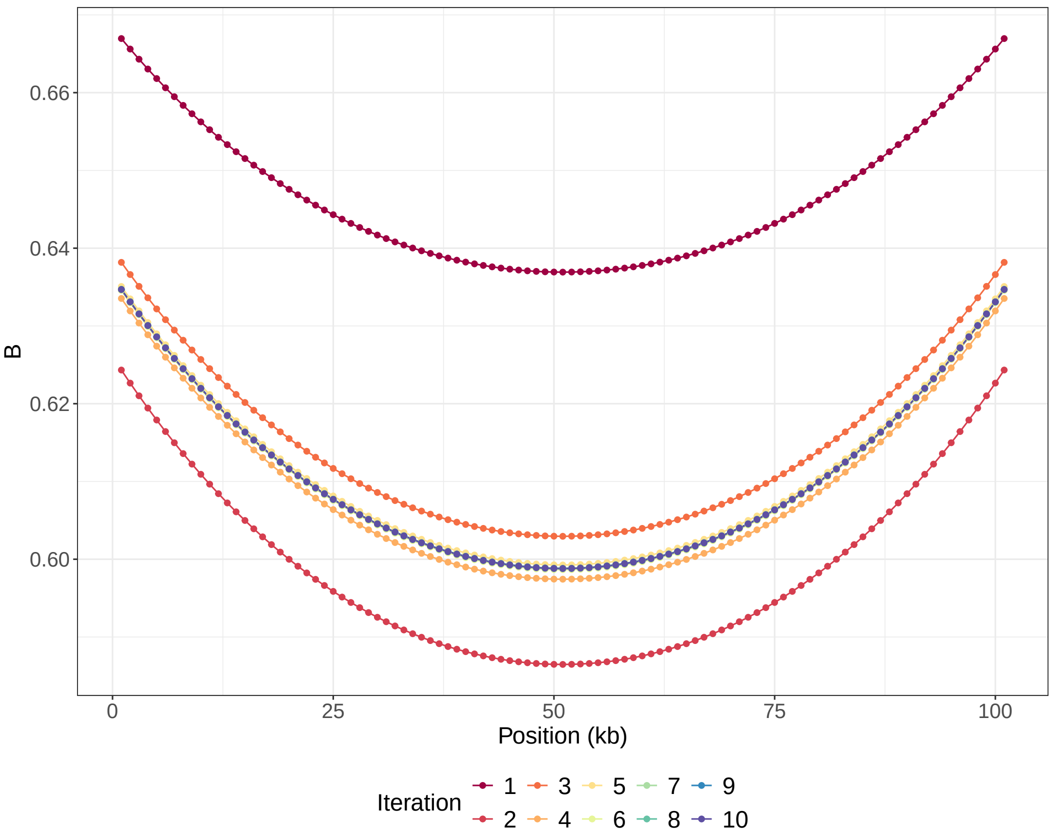

Figure S1: Predicted B-maps along a 100 kb segment, after each round of interference correction (color). Note how the iterative re-scaling of μ, r, and s leads to B-maps bouncing around the final values (convergence happens visually after the 6th iteration). Here the 100 kb segment layout follows that of Figure 2

bioRxiv preprint doi: https://doi.org/10.1101/2025.02.19.639084; this version posted September 16, 2025. The copyright holder for this preprint (which was not certified by peer review) is the author/funder, who has granted bioRxiv a license to display the preprint in perpetuity. It is made available under aCC-BY 4.0 International license.

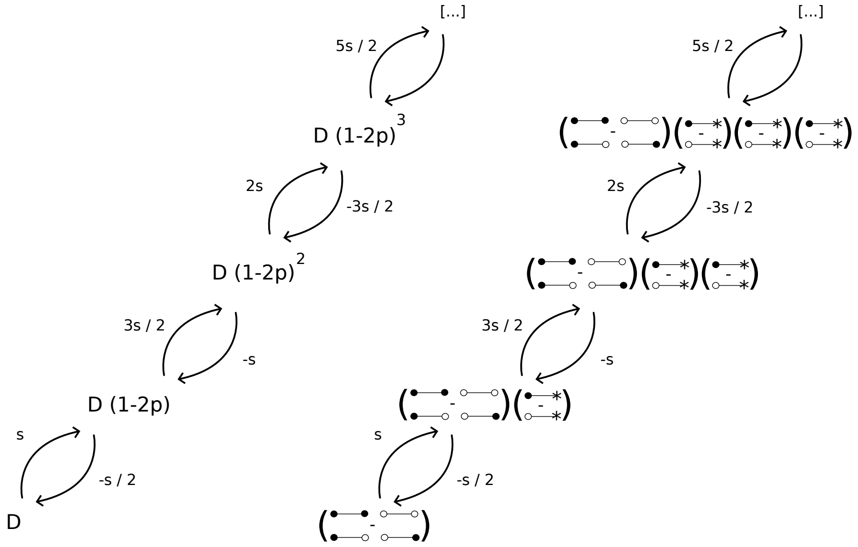

Figure S2: Representation of the dependencies among $D(1-2p)^j$ statistics imposed by the selection operator in (p, q, D)-space (left) and the equivalent haplotype space (right). Arrows denote "collects from", with the respective entry in the selection matrix shown above ($s < 0$). Haplotype subtractions proceed from top to bottom of the configuration within parentheses. Asterisks denote invariance to the allelic state at the right locus $(1-2p)$.

bioRxiv preprint doi: https://doi.org/10.1101/2025.02.19.639084; this version posted September 16, 2025. The copyright holder for this preprint (which was not certified by peer review) is the author/funder, who has granted bioRxiv a license to display the preprint in perpetuity. It is made available under aCC-BY 4.0 International license.

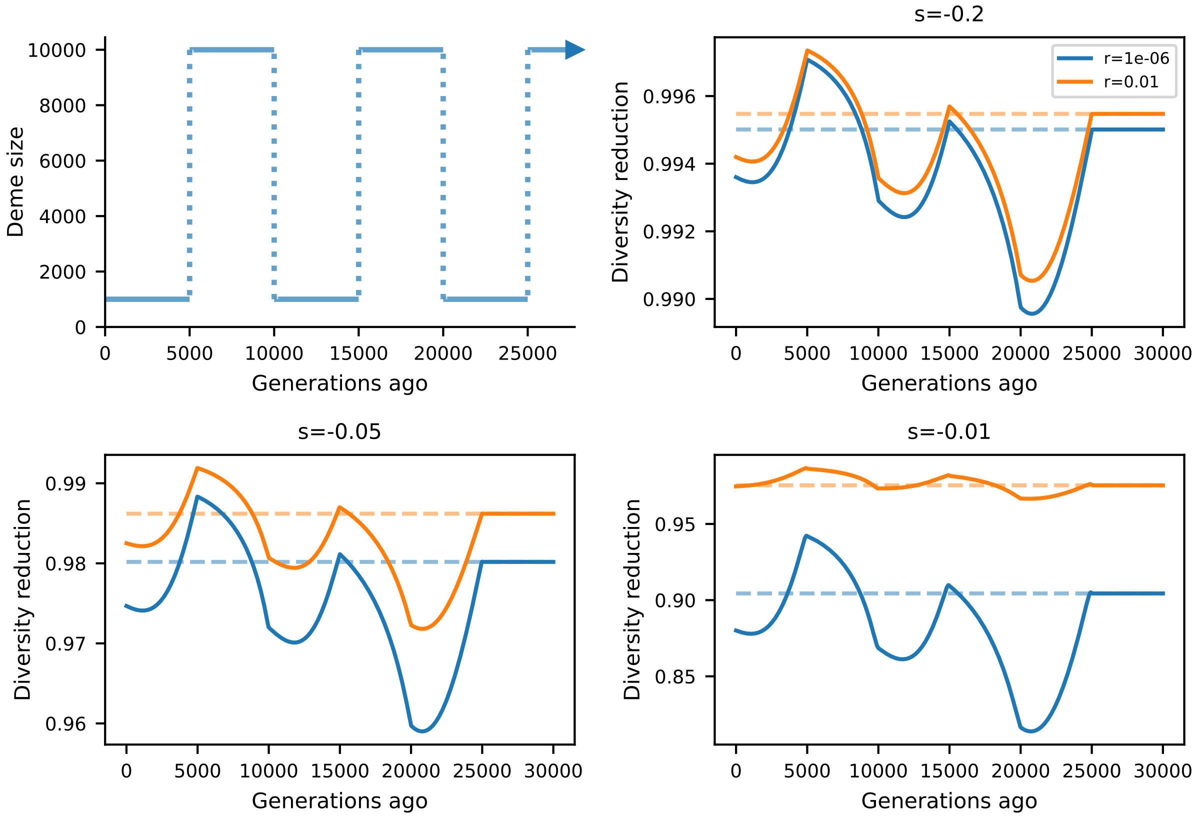

Figure S3: Temporal dynamics of BGS due to strong selection, as predicted using our extension to Nordborg (1997). Top left panel shows the demographic history considered. Each other panel shows, for a given selection coefficient, the predicted B-value trajectory for either tightly linked ($r = 10^{-6}$, blue lines) and loosely linked loci ($r = 10^{-2}$, orange lines). Dashed lines indicate the respective steady state solutions. Here the mutation rate is set to $u = 10^{-3}$.

bioRxiv preprint doi: https://doi.org/10.1101/2025.02.19.639084; this version posted September 16, 2025. The copyright holder for this preprint (which was not certified by peer review) is the author/funder, who has granted bioRxiv a license to display the preprint in perpetuity. It is made available under aCC-BY 4.0 International license.

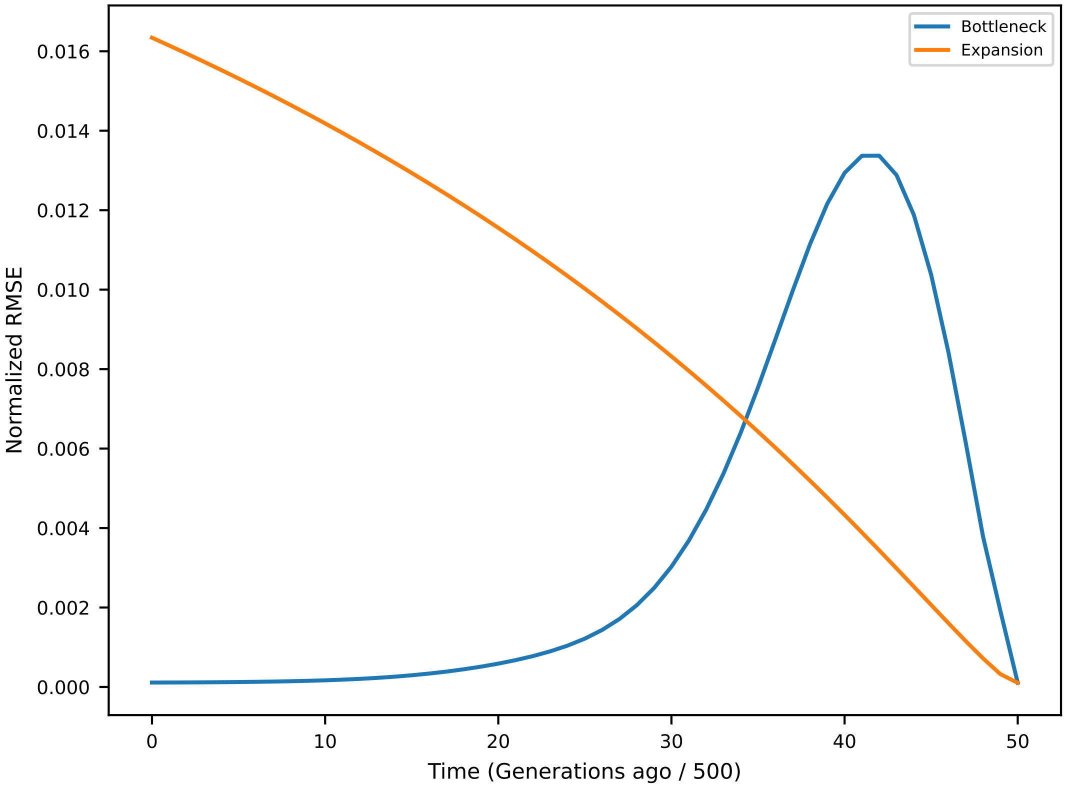

Figure S4: Temporal evolution of the the Normalized Rooted Mean Squared Error between the demography-aware B-map and steady-state B-maps, predicted every 500 generations after the size change. Blue and orange lines show results from a 10-fold bottleneck and expansion, respectively. Steady-state B-maps are obtained using the equilibrium N_e value calculated from neutral genetic diversity in the absence of linked selection (equivalent to the harmonic mean of population sizes in the relevant time-frame). Here the chromosome layout follows that of Figure 3.

bioRxiv preprint doi: https://doi.org/10.1101/2025.02.19.639084; this version posted September 16, 2025. The copyright holder for this preprint (which was not certified by peer review) is the author/funder, who has granted bioRxiv a license to display the preprint in perpetuity. It is made available under aCC-BY 4.0 International license.

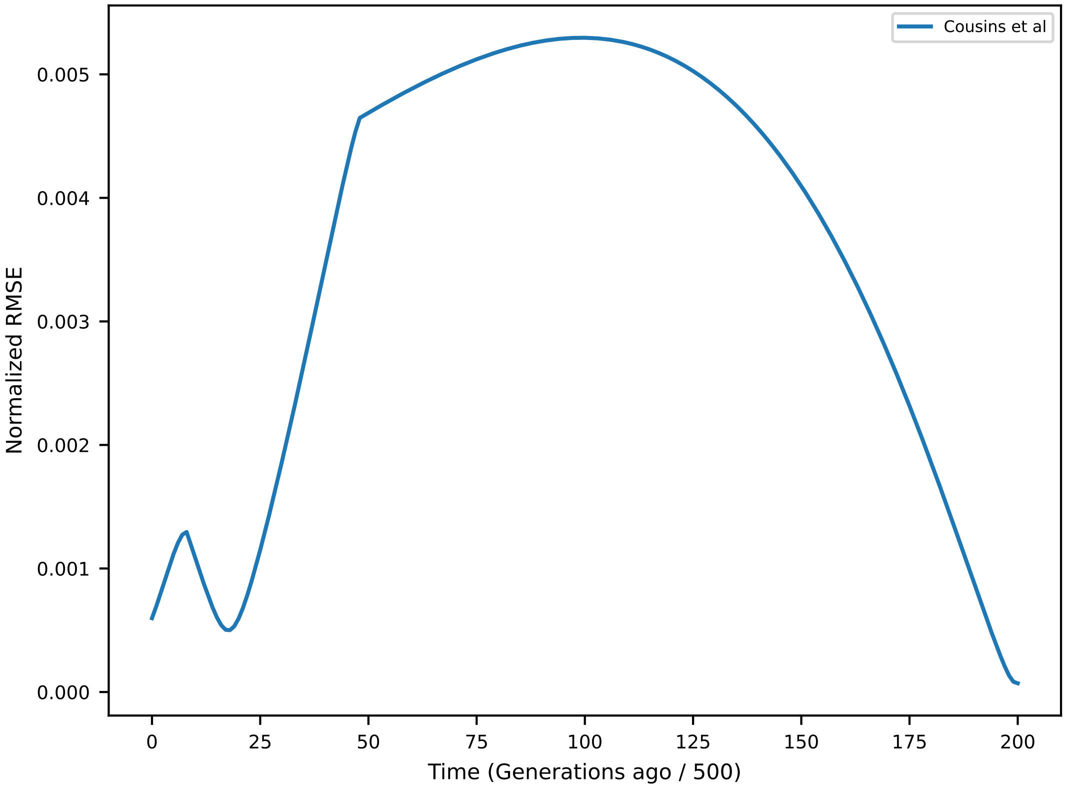

Figure S5: Temporal evolution of the the Normalized Rooted Mean Squared Error between the demography-aware B-map and steady-state B-maps, predicted every 500 generations after the first size change in the Cousins et al. (2024) demography. Steady-state B-maps are obtained using the equilibrium N_e value calculated from neutral genetic diversity in the absence of linked selection (equivalent to the harmonic mean of population sizes in the relevant time-frame). Here the chromosome layout follows that of Figure 3.

bioRxiv preprint doi: https://doi.org/10.1101/2025.02.19.639084; this version posted September 16, 2025. The copyright holder for this preprint (which was not certified by peer review) is the author/funder, who has granted bioRxiv a license to display the preprint in perpetuity. It is made available under aCC-BY 4.0 International license.

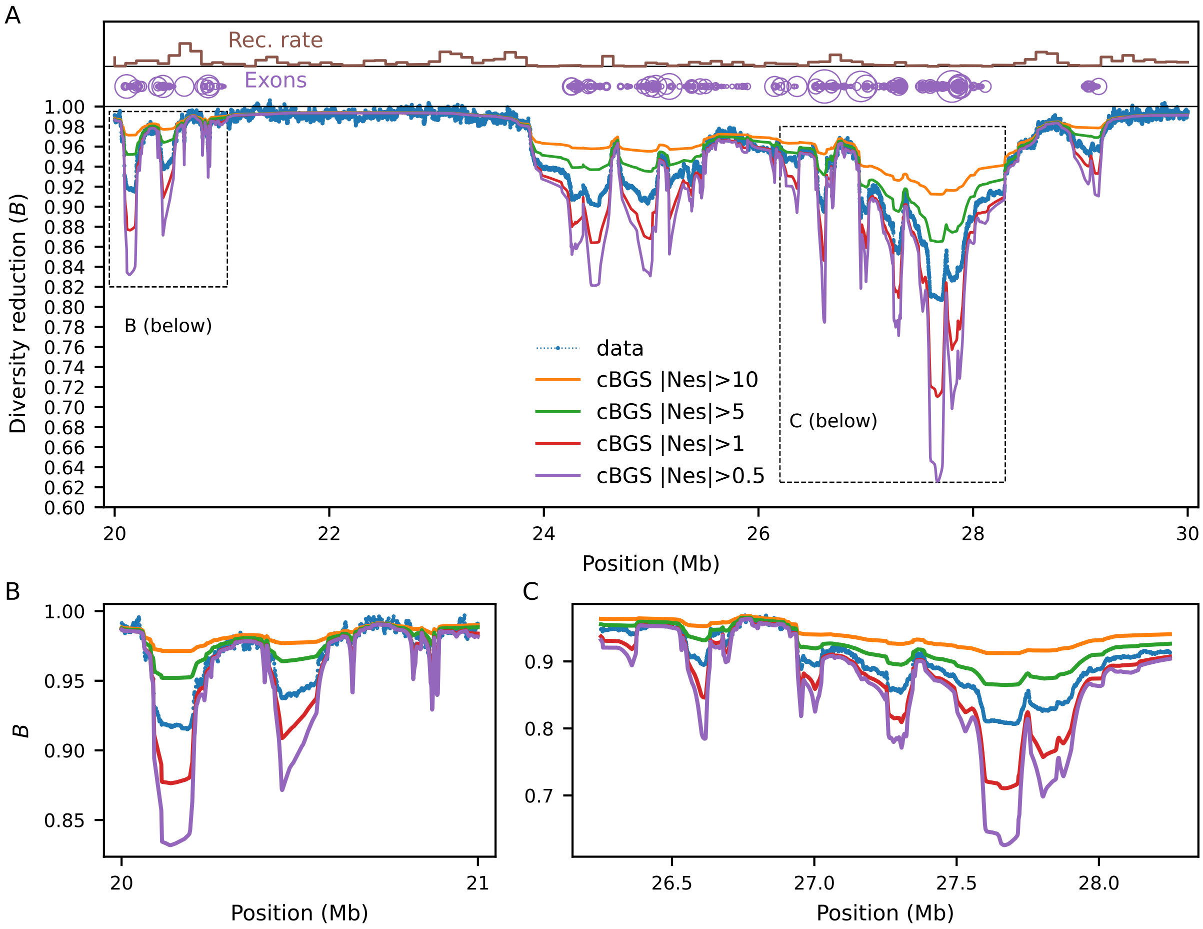

Figure S6: Predictions from cBGS, truncating the DFE at different points. Notice the overestimation of B-values caused by excluding a large portion of the DFE (orange and green lines) as well as the underestimation caused by the inappropriateness of the cBGS model at weaker selection coefficients (red and purple lines). Here the chromosome layout follows that of Figure 3.

bioRxiv preprint doi: https://doi.org/10.1101/2025.02.19.639084; this version posted September 16, 2025. The copyright holder for this preprint (which was not certified by peer review) is the author/funder, who has granted bioRxiv a license to display the preprint in perpetuity. It is made available under aCC-BY 4.0 International license.

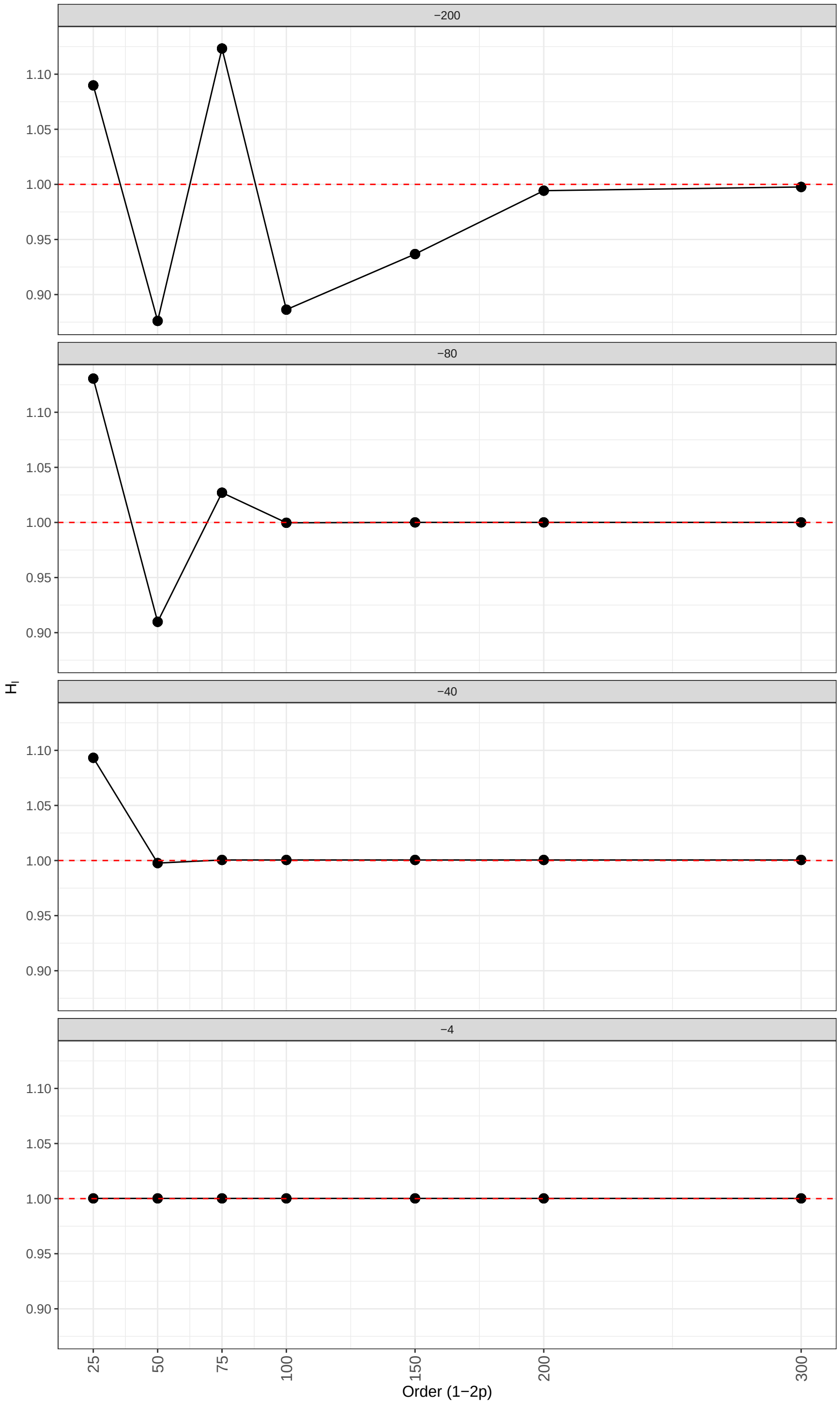

Figure S7: Benchmarking of the implementation of the selection operator in `moments++`, with the truncation strategy to close the system. Shown are ratios of π_l predicted with `moments++` to π_l predicted with `moments.TwoLocus`, as a function of the Order of $1 - 2p$ factors included in the `moments++` model (panels show $N_e s$). Dashed red lines denote the target line indicating good agreement with our gold-standard.

bioRxiv preprint doi: https://doi.org/10.1101/2025.02.19.639084; this version posted September 16, 2025. The copyright holder for this preprint (which was not certified by peer review) is the author/funder, who has granted bioRxiv a license to display the preprint in perpetuity. It is made available under aCC-BY 4.0 International license.

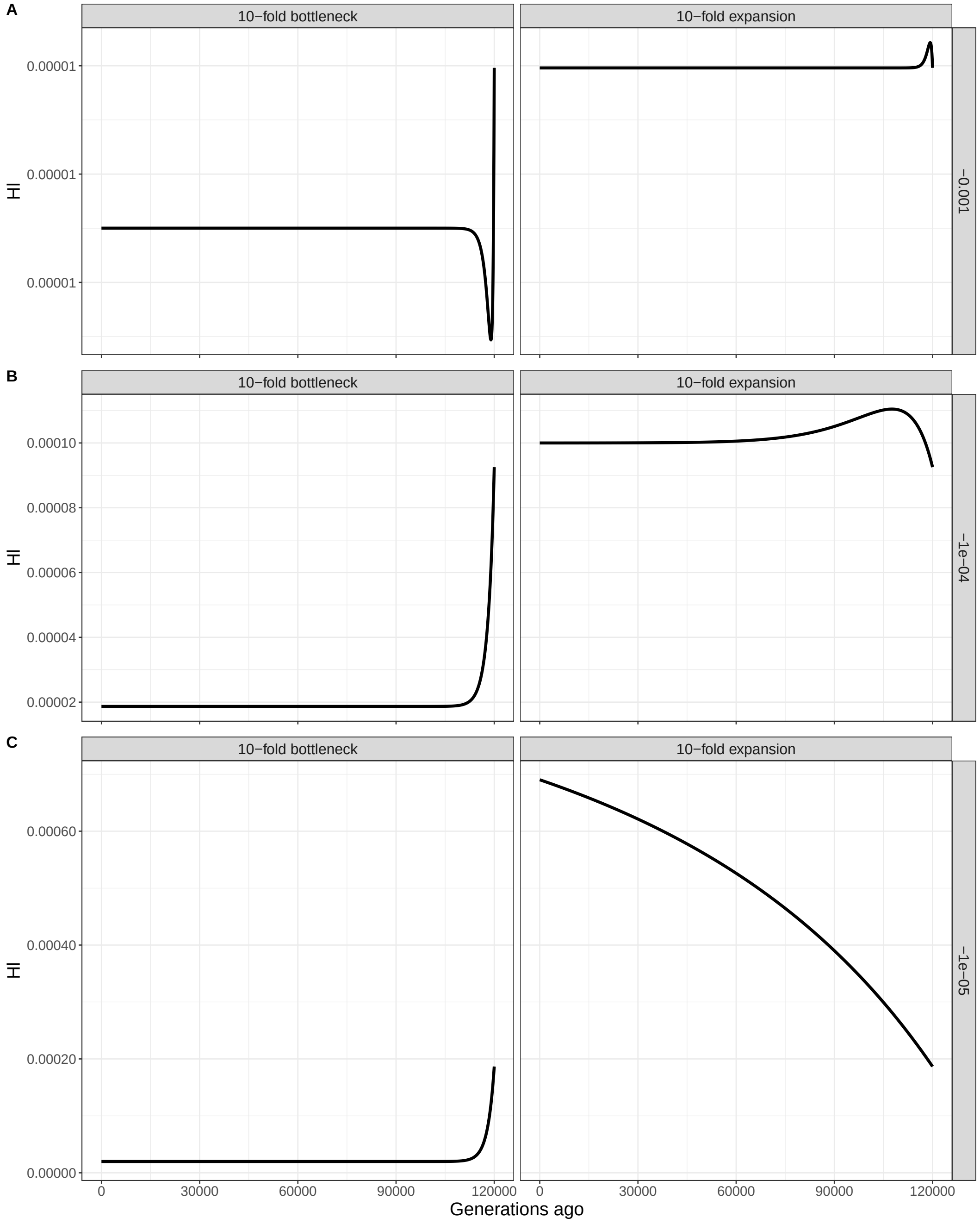

Figure S8: Temporal trajectories of π_L following a 10-fold bottleneck (left panels) or expansion (right panels). A) $s = -0.001$. B) $s = -0.0001$. C) $s = -0.00001$. Here $\mu = 10^{-8}$.

bioRxiv preprint doi: https://doi.org/10.1101/2025.02.19.639084; this version posted September 16, 2025. The copyright holder for this preprint (which was not certified by peer review) is the author/funder, who has granted bioRxiv a license to display the preprint in perpetuity. It is made available under aCC-BY 4.0 International license.

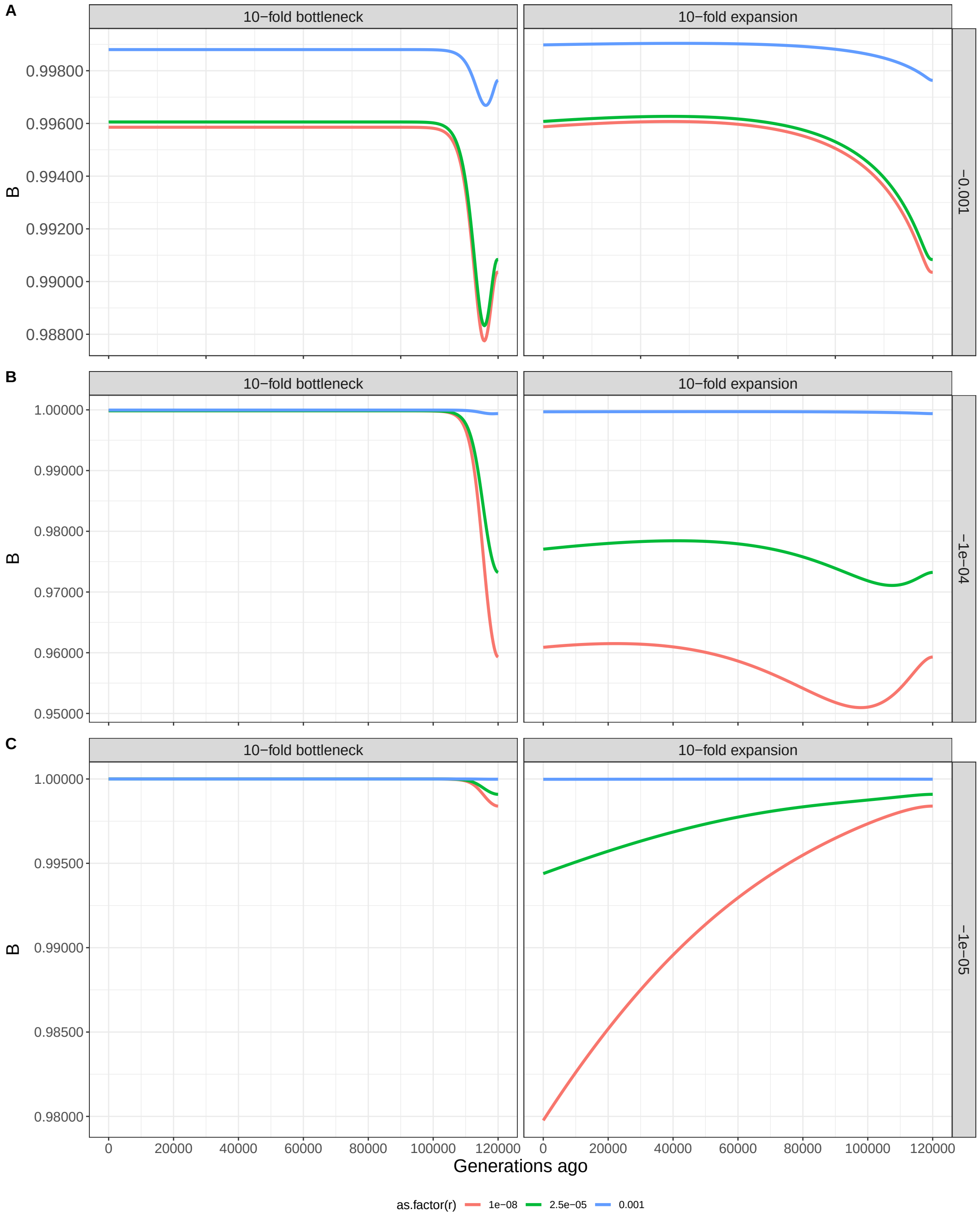

Figure S9: Temporal trajectories of B-values following a 10-fold bottleneck (left panels) or expansion (right panels), for a pure two-locus model. Note how the expansion scenario does not reach the new equilibrium after 120,000 generations. A) $s = -0.001$. B) $s = -0.0001$. C) $s = -0.00001$. Colors denote the rate of recombination. Here $\mu = 10^{-8}$ and B-values are raised to the 1000th power to facilitate visualization.

bioRxiv preprint doi: https://doi.org/10.1101/2025.02.19.639084; this version posted September 16, 2025. The copyright holder for this preprint (which was not certified by peer review) is the author/funder, who has granted bioRxiv a license to display the preprint in perpetuity. It is made available under aCC-BY 4.0 International license.

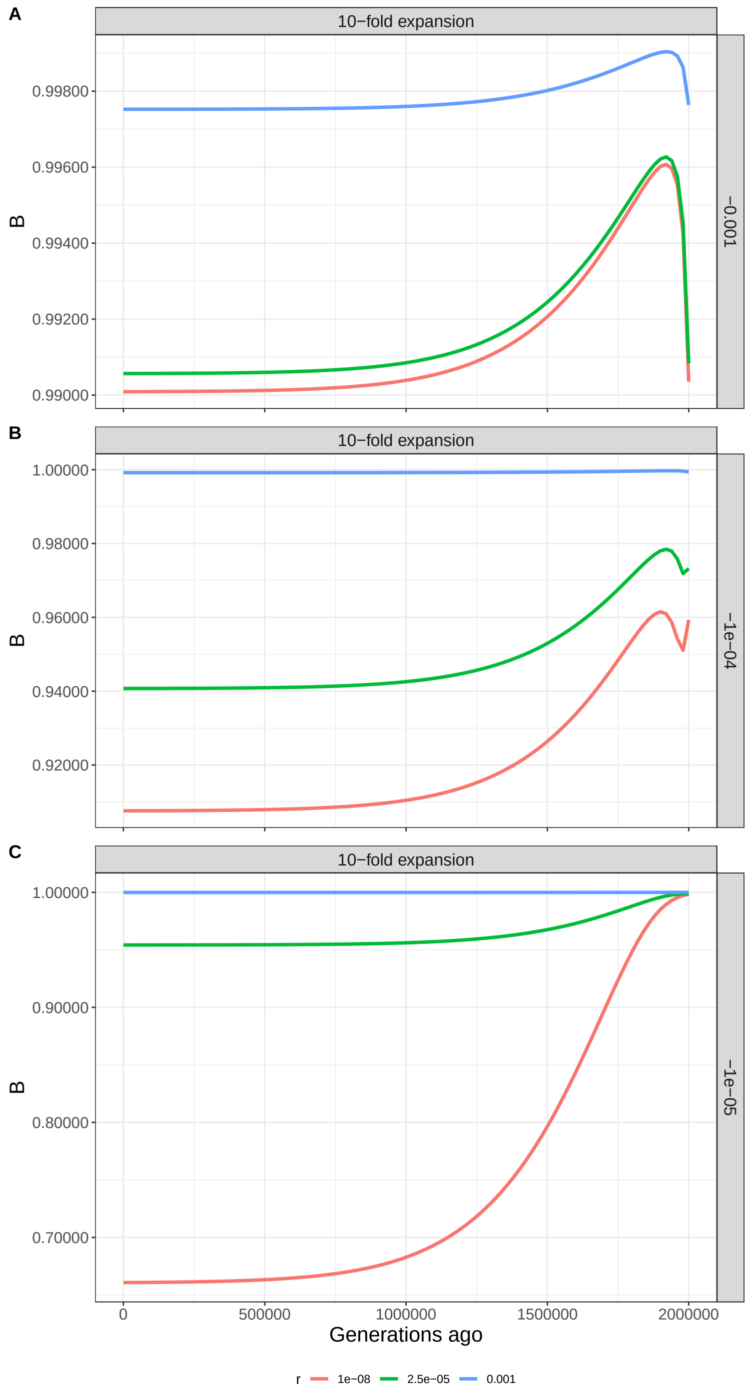

Figure S10: Temporal trajectories of B-values under the 10-fold expansion model from Figure S9 where we track statistics for 2,000,000 generations to ensure that the new equilibrium point is found. A) $s = -0.001$. B) $s = -0.0001$. C) $s = -0.00001$. Colors denote the rate of recombination. Here $\mu = 10^{-8}$ and B-values are raised to the 1000th power to facilitate visualization.

bioRxiv preprint doi: https://doi.org/10.1101/2025.02.19.639084; this version posted September 16, 2025. The copyright holder for this preprint (which was not certified by peer review) is the author/funder, who has granted bioRxiv a license to display the preprint in perpetuity. It is made available under aCC-BY 4.0 International license.

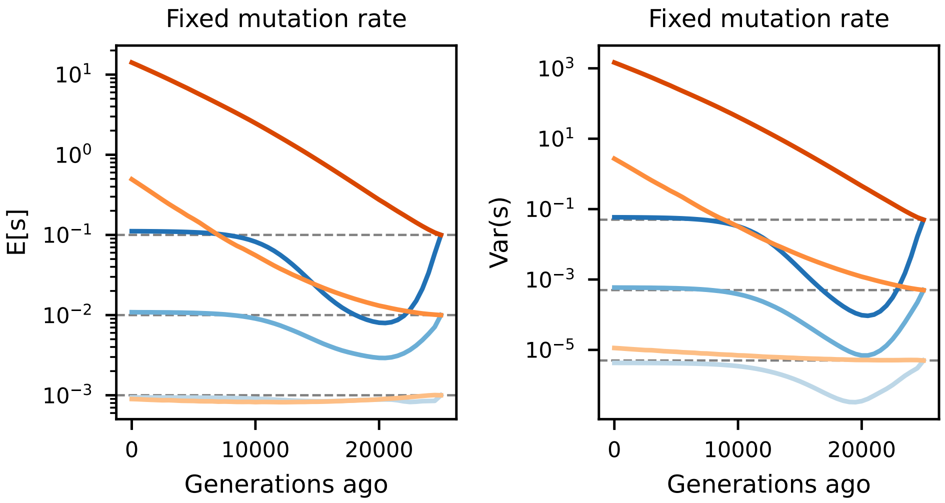

Figure S11: Mean (left) and variance (right) of the inferred DFEs depicted in Figure 4.

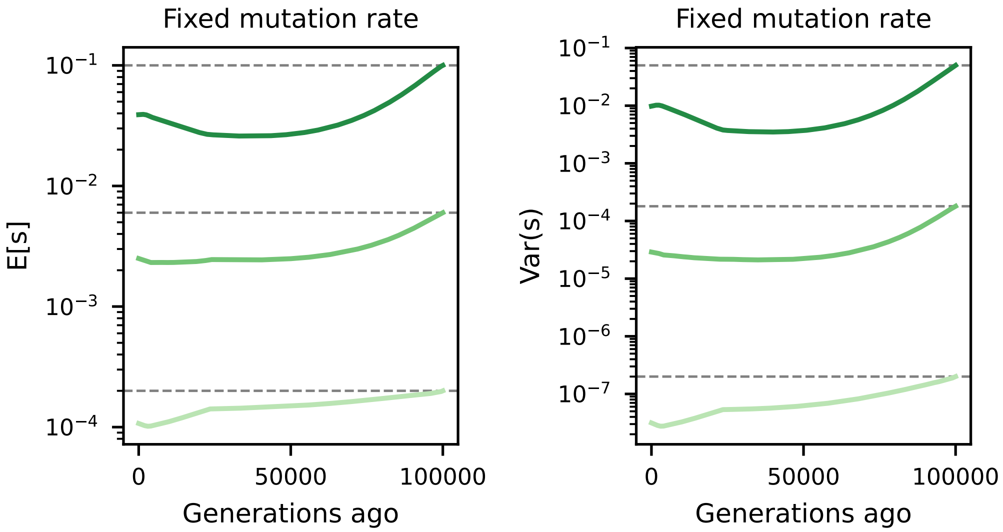

Figure S12: Mean (left) and variance (right) of the inferred DFEs depicted in Figure 5.
