# Supplementary material for: A model for background selection in non-equilibrium populations
Source: bioRxiv. 2025 Feb 20:2025.02.19.639084. Preprint. [Version 1] doi: 10.1101/2025.02.19.639084 (PMC11870586; doi:10.1101/2025.02.19.639084)
Supplement: Supplementary file 1 [file NIHPP2025.02.19.639084V1-supplement-1.pdf]

bioRxiv preprint doi: https://doi.org/10.1101/2025.02.19.639084; this version posted February 21, 2025. The copyright holder for this preprint (which was not certified by peer review) is the author/funder, who has granted bioRxiv a license to display the preprint in perpetuity. It is made available under aCC-BY 4.0 International license.

Supplemental Figures

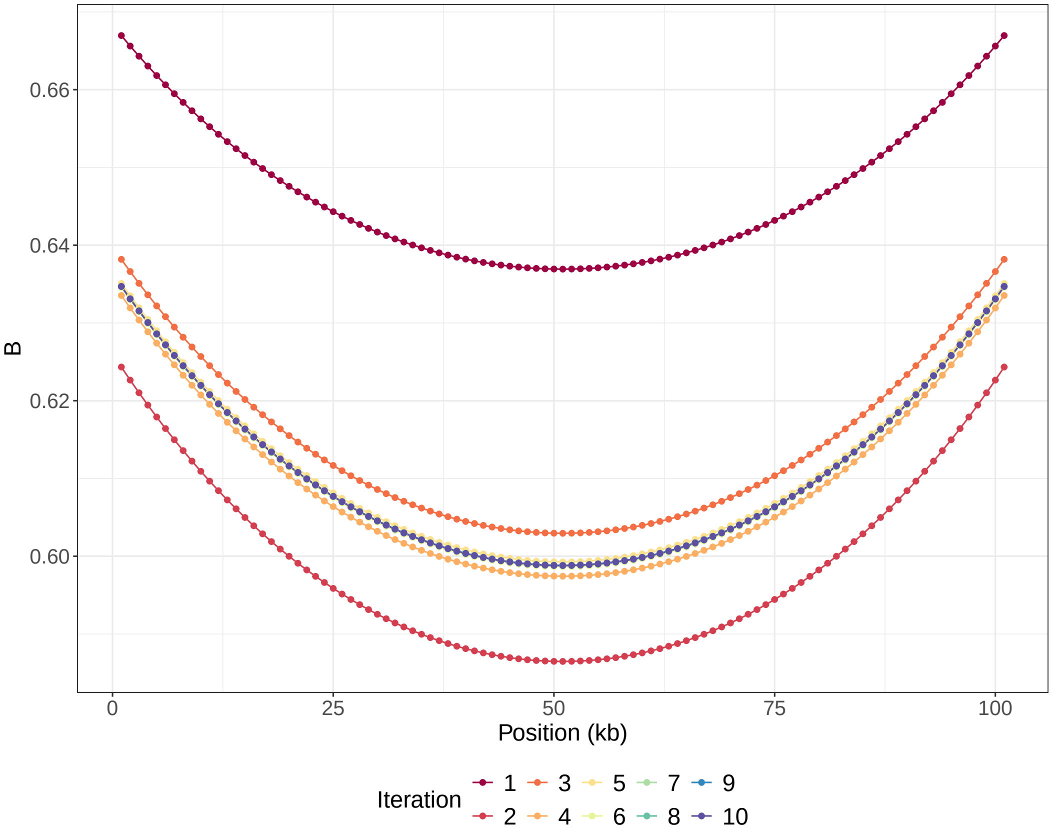

Figure S1: Predicted B-maps after each round of interference correction. Convergence happens visually after the 6th iteration. Here the 100 kb segment layout follows that of Figure 2

bioRxiv preprint doi: https://doi.org/10.1101/2025.02.19.639084; this version posted February 21, 2025. The copyright holder for this preprint (which was not certified by peer review) is the author/funder, who has granted bioRxiv a license to display the preprint in perpetuity. It is made available under aCC-BY 4.0 International license.

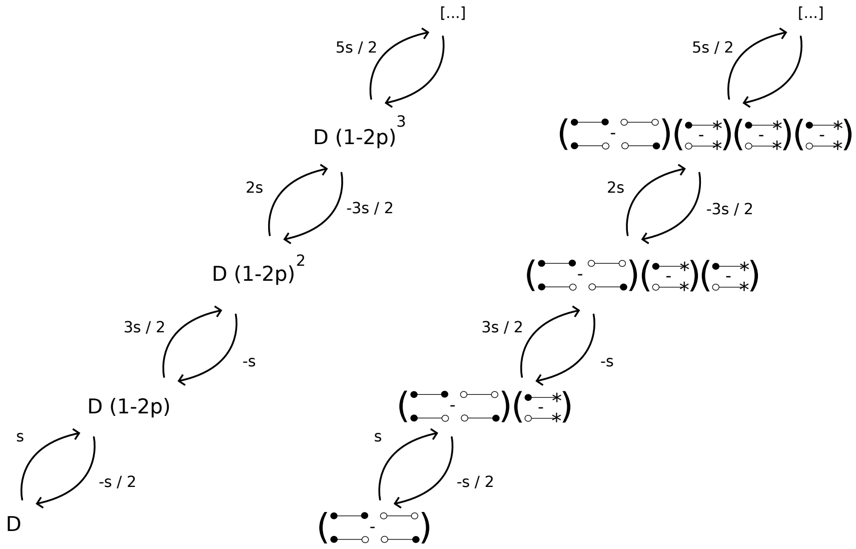

Figure S2: Representation of the dependencies among $D(1-2p)^j$ statistics imposed by the selection operator in (p, q, D)-space (left) or haplotype space (right). Haplotype subtractions proceed from top to bottom of the configuration within parentheses. Asterisks denote invariance to the allelic state at the right locus $(1-2p)$.

bioRxiv preprint doi: https://doi.org/10.1101/2025.02.19.639084; this version posted February 21, 2025. The copyright holder for this preprint (which was not certified by peer review) is the author/funder, who has granted bioRxiv a license to display the preprint in perpetuity. It is made available under aCC-BY 4.0 International license.

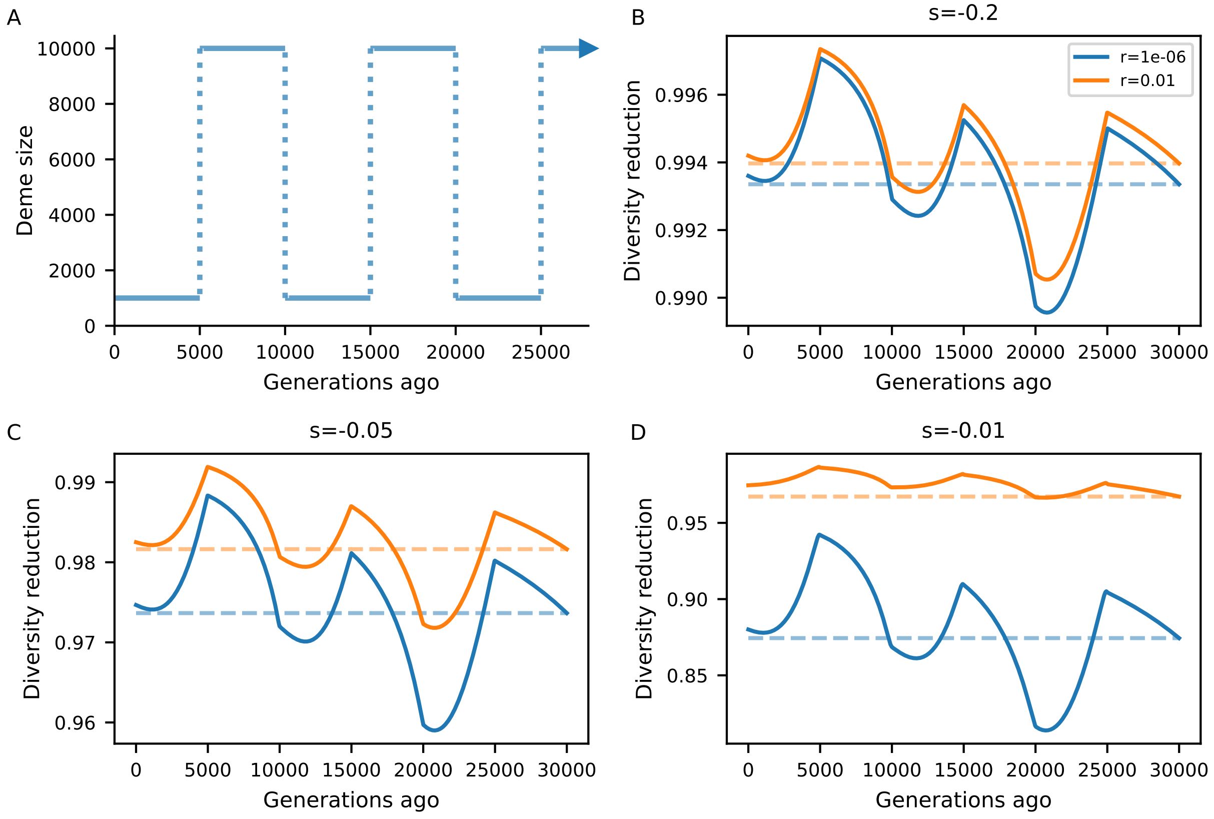

Figure S3: Dynamics of BGS due to strong selection, as predicted using our extension to Nordborg (1997). For each selection coefficient, we consider tightly linked ($r = 10^{-6}$) and loosely linked loci ($r = 10^{-2}$). The mutation rate is set to $u = 10^{-3}$. Dashed lines indicate the steady state solution.

bioRxiv preprint doi: https://doi.org/10.1101/2025.02.19.639084; this version posted February 21, 2025. The copyright holder for this preprint (which was not certified by peer review) is the author/funder, who has granted bioRxiv a license to display the preprint in perpetuity. It is made available under aCC-BY 4.0 International license.

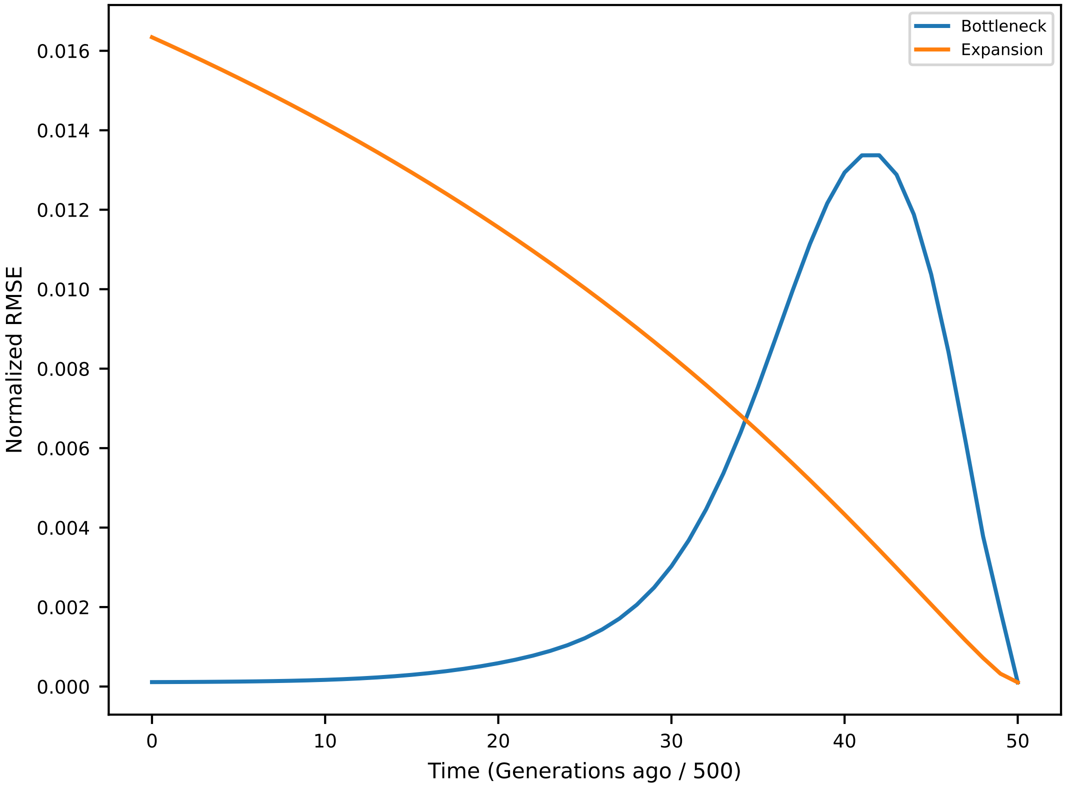

Figure S4: Temporal evolution of the the Normalized Rooted Mean Squared Error between the demography-aware B-map and steady-state B-maps, predicted every 500 generations after the 10-fold size change. Here the chromosome layout follows that of Figure 3.

bioRxiv preprint doi: https://doi.org/10.1101/2025.02.19.639084; this version posted February 21, 2025. The copyright holder for this preprint (which was not certified by peer review) is the author/funder, who has granted bioRxiv a license to display the preprint in perpetuity. It is made available under aCC-BY 4.0 International license.

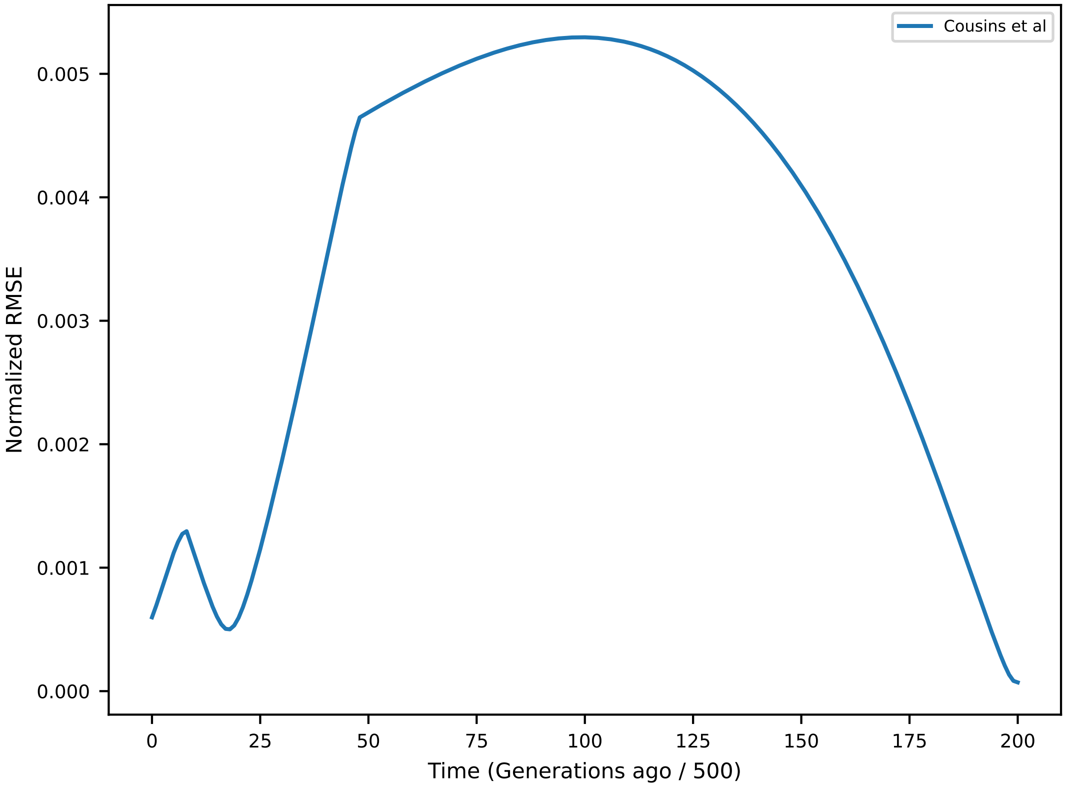

Figure S5: Temporal evolution of the the Normalized Rooted Mean Squared Error between the demography-aware B-map and steady-state B-maps, predicted every 500 generations after the first size change in the Cousins et al. (2024) demography. Here the chromosome layout follows that of Figure 3.

bioRxiv preprint doi: https://doi.org/10.1101/2025.02.19.639084; this version posted February 21, 2025. The copyright holder for this preprint (which was not certified by peer review) is the author/funder, who has granted bioRxiv a license to display the preprint in perpetuity. It is made available under aCC-BY 4.0 International license.

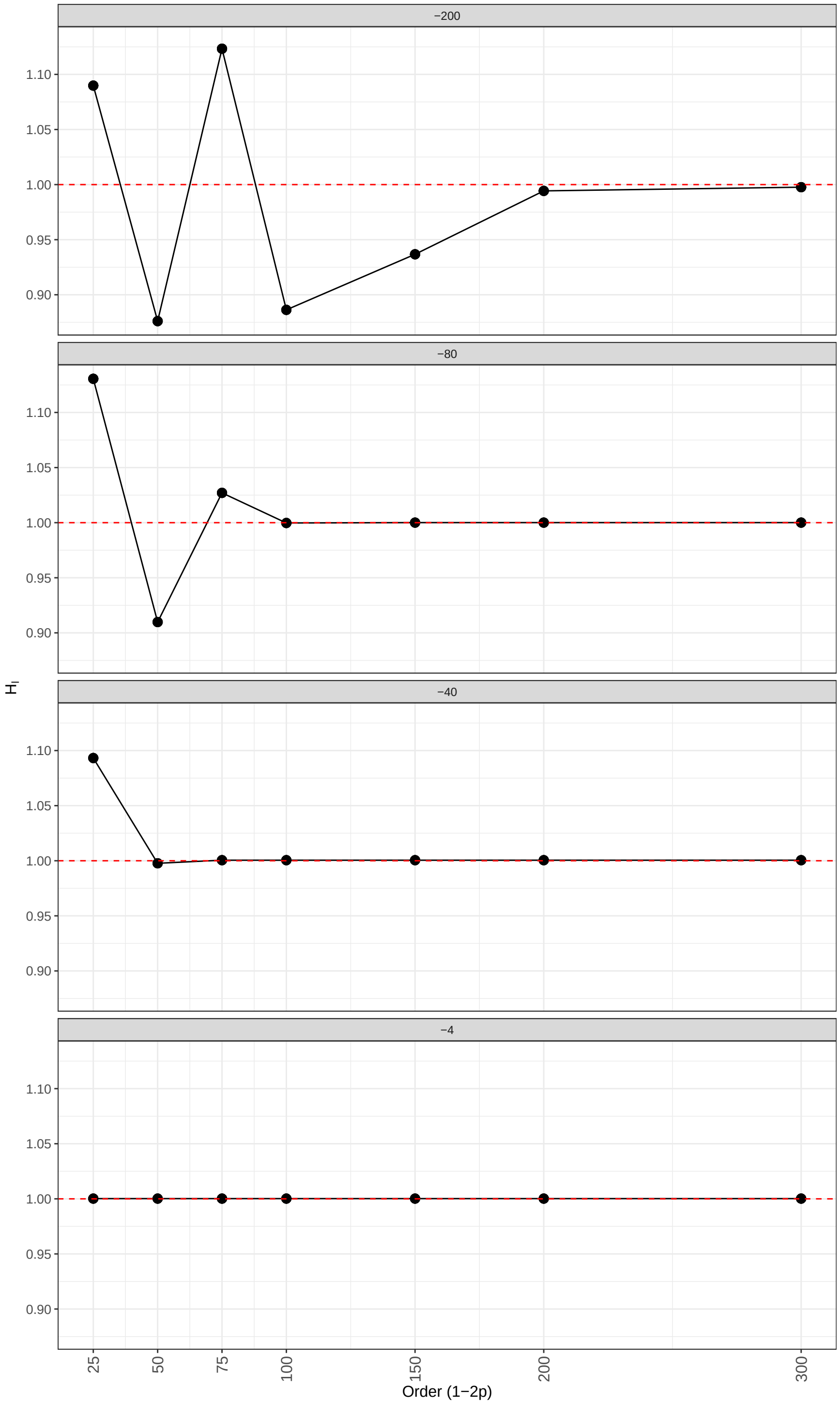

Figure S6: π_l predicted with `moments++` divided by π_l predicted with `moments.TwoLocus`, as a function of the Order of $1 - 2p$ factors included in the model and the efficiencay of selection (panels show $N_e s$).

bioRxiv preprint doi: https://doi.org/10.1101/2025.02.19.639084; this version posted February 21, 2025. The copyright holder for this preprint (which was not certified by peer review) is the author/funder, who has granted bioRxiv a license to display the preprint in perpetuity. It is made available under aCC-BY 4.0 International license.

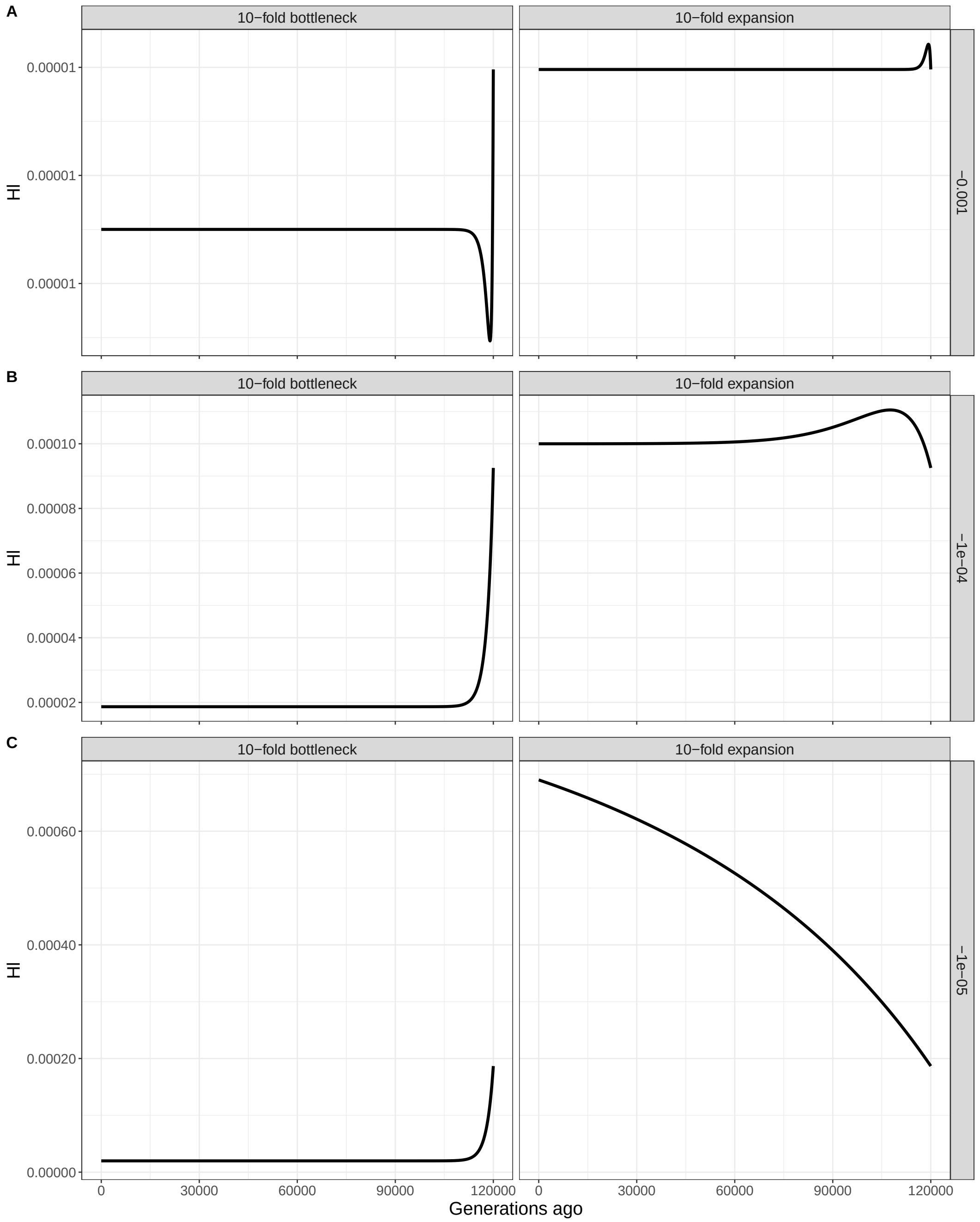

Figure S7: Temporal trajectories of π_L as a function of s and the two-epoch demography. Here $\mu = 10^{-8}$.

bioRxiv preprint doi: https://doi.org/10.1101/2025.02.19.639084; this version posted February 21, 2025. The copyright holder for this preprint (which was not certified by peer review) is the author/funder, who has granted bioRxiv a license to display the preprint in perpetuity. It is made available under aCC-BY 4.0 International license.

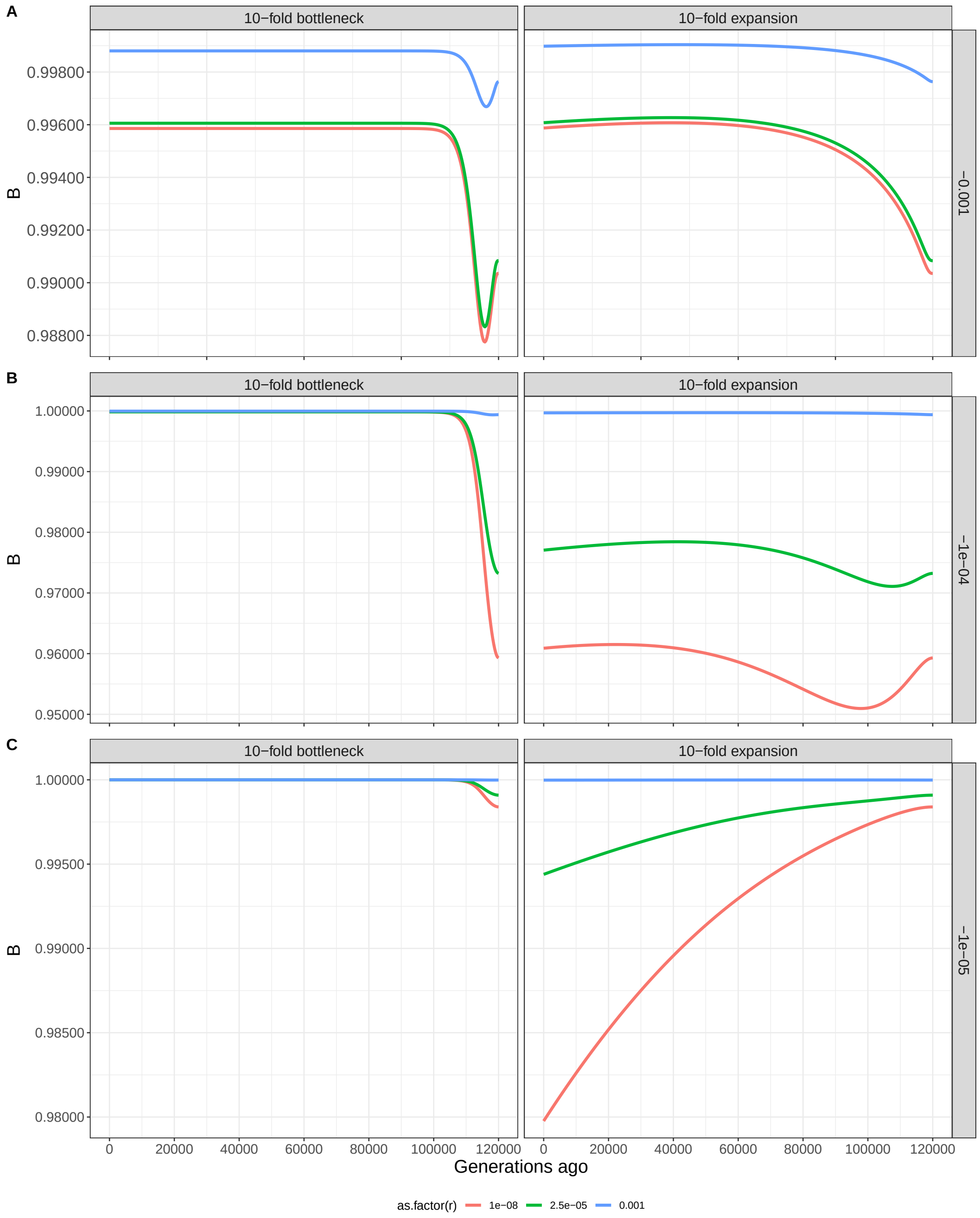

Figure S8: Temporal trajectories of B-values as a function of s and the two-epoch demography. A) $s = -0.001$. B) $s = -0.0001$. C) $s = -0.00001$. Colors denote the rate of recombination with the constrained locus. Here $\mu = 10^{-8}$ and B-values are raised to the 1000th power to facilitate visualization.

bioRxiv preprint doi: https://doi.org/10.1101/2025.02.19.639084; this version posted February 21, 2025. The copyright holder for this preprint (which was not certified by peer review) is the author/funder, who has granted bioRxiv a license to display the preprint in perpetuity. It is made available under aCC-BY 4.0 International license.

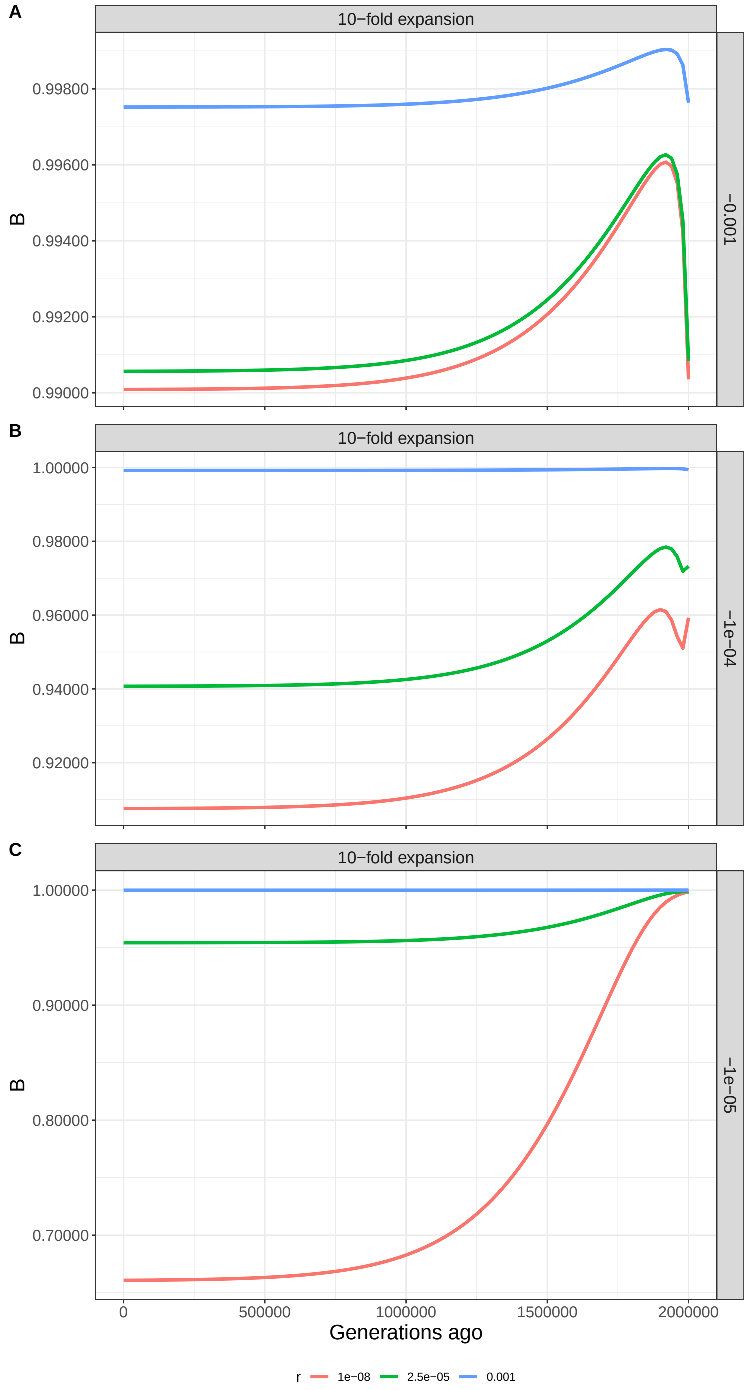

Figure S9: Temporal trajectories of B-values under the 10x expansion model from Figure S8 where we track statistics for 2,000,000 generations to ensure that the new equilibrium point is found. A) $s = -0.001$. B) $s = -0.0001$. C) $s = -0.00001$. Colors denote the rate of recombination with the constrained locus. Here $\mu = 10^{-8}$ and B-values are raised to the 1000th power to facilitate visualization.

bioRxiv preprint doi: https://doi.org/10.1101/2025.02.19.639084; this version posted February 21, 2025. The copyright holder for this preprint (which was not certified by peer review) is the author/funder, who has granted bioRxiv a license to display the preprint in perpetuity. It is made available under aCC-BY 4.0 International license.

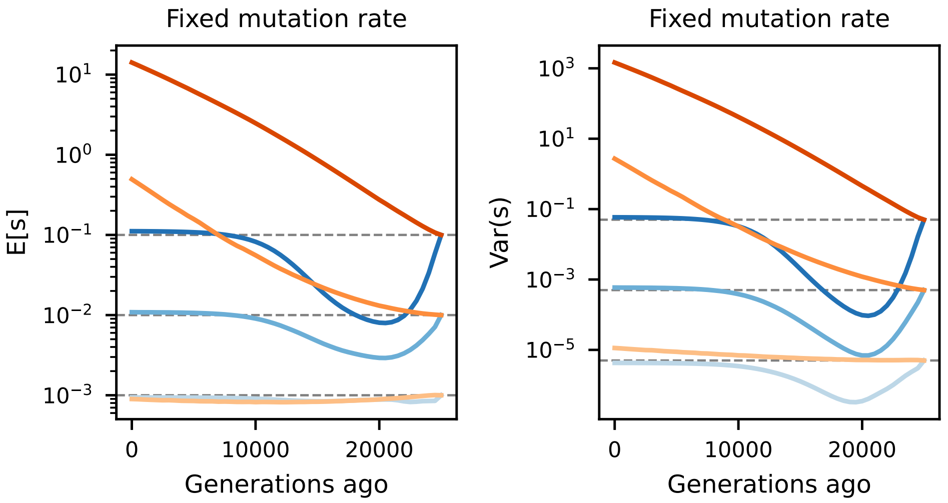

Figure S10: Mean (left) and variance (right) of the inferred DFEs depicted in Figure 4.

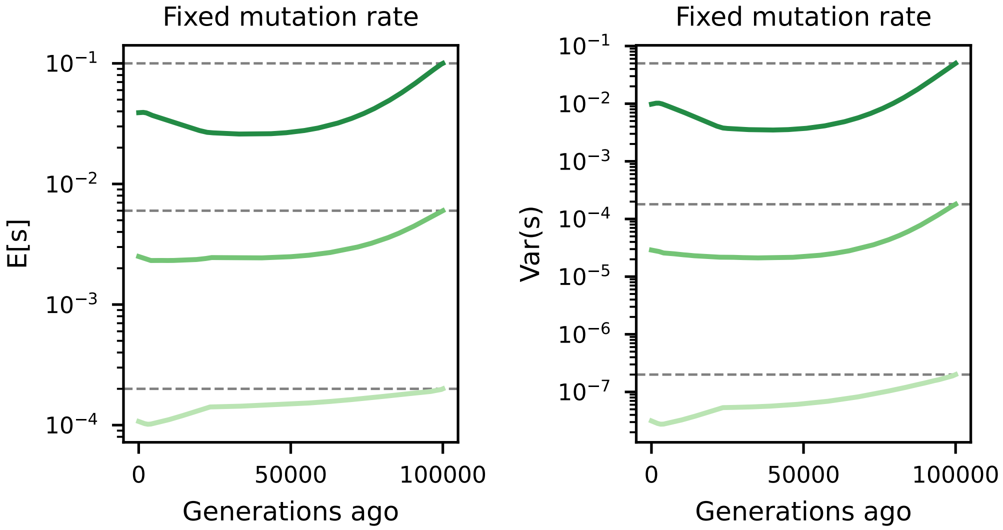

Figure S11: Mean (left) and variance (right) of the inferred DFEs depicted in Figure 5.
